# Comparison of posterior cruciate retention and substitution in total knee arthroplasty during gait: a systematic review and meta-analysis

**DOI:** 10.1186/s13018-022-03047-y

**Published:** 2022-03-09

**Authors:** Chunjiang Li, Mingjie Dong, Dinglong Yang, Zhiqiang Zhang, Junjun Shi, Ruipeng Zhao, Xiaochun Wei

**Affiliations:** 1 Department of Orthopaedics, The Second Hospital of Shanxi Medical University, No. 382 Wuyi Road, Taiyuan, 030001 China; 2 Department of Orthopaedics, The Second Clinical Medical College of Shanxi Medical University, Taiyuan, 030001 China

**Keywords:** Gait, Posterior cruciate retention, Posterior cruciate substitution, Total knee arthroplasty

## Abstract

**Background:**

To compare the gait patterns between posterior cruciate retention and substitution in total knee arthroplasty (TKA).

**Methods:**

Electronic databases including the PubMed, Embase, CINAHL, Web of Science, and Cochrane databases were searched to identify clinical trials investigating posterior cruciate retention versus substitution in TKA. The outcome measurements were the kinematic gait parameters (flexion at heel strike, maximum flexion during loading response, flexion range during loading, minimal flexion at terminal stance, maximal flexion at the swing, and total flexion during the gait cycle), Knee Society Score (KSS), knee flexion, knee extension, and walking speed. Statistical software Review Manager 5.4 and Stata 14.0 were used for data analysis.

**Results:**

There were finally 9 studies included in this meta-analysis. The results did not reveal differences between posterior cruciate retention (CR) and posterior cruciate substitution (PS) groups in TKA, in terms of kinematic gait parameters, knee extension, walking speed, and KSS. However, the PS group had a significantly larger knee flexion angle than that in the CR group [weighted mean difference = − 3.20, 95% CI − 6.13 to − 0.28, *P* = 0.03].

**Conclusion:**

Both the posterior cruciate retention and posterior cruciate substitution lead to obvious improvements in patient function and have their advantages in getting a good cup position. The PS design is significantly better on the knee flexion, while there are no statistical differences in kinematic gait parameters and outcome scores between them. This might indicate that surgeons do not necessarily need a PS design to substitute the posterior cruciate ligament during TKA.

## Background

The knee joint is one of the largest, most complex, and most important joints in the human body. But the joint function and quality of life in people are seriously affected with the incidence of knee osteoarthritis increasing all over the world. Osteoarthritis is a degenerative joint disease that leads to the degradation of articular cartilage and subchondral bone [1]. Clinically, patients with knee osteoarthritis are generally characterized by impaired knee function and disabling knee pain. In the later stage of the disease, the only remaining treatment is total knee arthroplasty, which is a common and effective surgical operation to relieve permanent pain. The total knee arthroplasty is to resurface the joint articulating surfaces. The posterior cruciate ligament involved in the knee joint is commonly either retained or replaced by artificial structures during total knee arthroplasty surgery, i.e., posterior cruciate retention and posterior cruciate substitution. Several randomized studies comparing two designs have been conducted from the early 90 s up to now [2, 3], but the debate continues today in terms of the significance of preserving the posterior cruciate ligament (PCL) in total knee arthroplasty surgery. It is generally assumed that CR design could increase range of motion and knee flexion by restoring anatomical femoral rollback and normal knee biomechanics, but some studies show a lack of posterior femorotibial translation with knee flexion in CR design [4, 5]. Besides, several studies also show that preservation of the posterior cruciate ligament in TKA surgery does not guarantee the proper function of this ligament [6, 7]. The PS design has a cam post mechanism to substitute for the PCL and permits rollback of the femoral component on the tibial component during flexion [1]. And its proponents argue that the posterior translation of the femur creates more clearance on the tibia, and theoretically, more knee flexion [8]. In general, numerous studies have reported that both designs show satisfactory results, but the specific importance of posterior cruciate ligament retention has yet to be confirmed, and the particular advantages of one design over the other have not been documented.

In addition, some studies have shown no difference between CR and PS designs in knee flexion and kinematic gait parameters [8–10]. However, others have found a marked improvement in PS design concerning knee flexion and range of motion [11–13], including one systematic review [14]. These contradictory results hinder consensus. Therefore, the meta-analysis was designed to mainly compare knee flexion and kinematic gait parameters of CR and PS designs by accumulating data of the included studies, thus more accurately assessing the differences and providing more theoretical guidance for clinical practice. In addition, this analysis attempted to analyze the clinical and functional results of treatment between the two designs with the Knee Society Score (KSS), extension, and walking speed, as well.

## Methods

The meta-analysis was performed according to the Preferred Reporting Items for Systematic Reviews and Meta-Analyses (PRISMA) statement guidelines.

### Search strategy

According to the recommendations of the Cochrane Collaborations, multiple comprehensive databases were retrieved for studies, including PubMed, Embase, CINAHL, Web of Science, and Cochrane Library from January 1990 to November 2021. Endeavoring to find the gray literature by searching the magazine catalog and references manually. All of the relevant papers were searched without language restrictions and translated if necessary. The search was conducted by using the keywords, “gait,” “total knee arthroplasty,” “TKA,” “posterior cruciate retention,” and “posterior cruciate substitution.” The search strategy was: gait AND cruciate-retaining OR cruciate retention AND stabilized OR substitute* AND total knee arthroplasty OR TKA. Manually searching pertinent papers and their bibliographies after the initial search.

### Study selection

Two reviewers independently selected the pertinent studies from the title and abstract to conduct a comprehensive review. When the abstract did not provide sufficient data, the full text of the study was reviewed. The studies meeting set criteria were identified and included. The inclusion criteria were (1) patients with one or both knees degenerative joint diseases of any gender, age, or race; (2) patients who underwent for TKA with CR or PS prostheses; (3) studies compared the postoperative gait patterns; (4) retrospective studies, prospective studies, controlled trials, and cohort studies; (5) reported in detail the number of subjects in CR and PS groups, as well as the mean and standard deviation of parameters; and (6) used appropriate statistical methods to compare parameters between groups. The exclusion criteria for the study were (1) duplicate publications; (2) cadaveric studies; (3) case reports, reviews, letters, editorials, commentaries, and expert opinions; (4) studies with incomplete or missing outcome data; (5) failed to meet the inclusion criteria in terms of studies objectives and interventions; and (6) the original document of imprecise experimental design.

### Data extraction and quality assessment

According to the pre-established data extraction form, two investigators (M.-J.D. and D.-L.Y.) independently extracted the data in all included studies and resolved any disagreements between them by consulting a third reviewer (C.-J.L.). The information extracted included: (1) the basic characteristics of the included papers, including the article title, authors, publication date, journal title and volume, etc.; (2) methodological characteristics of the research: blinded, randomized, and controlled, etc.; (3) the patients’ related characteristics, including age, gender, race, disease course and severity, etc.; and (4) sample size, study type, intervention methods, follow-up time, and outcome measurements, etc. When the published information was unclear for the analysis, communication with the authors of published eligible studies was attempted. The risk-of-bias assessment tool outlined in Cochrane Handbook was used to assess the quality of methodology in controlled clinical trials; six domains were evaluated: (1) random sequence generation, (2) allocation concealment, (3) blinding of patients and personnel, (4) blinding of outcome assessment, (5) incomplete outcome data, and (6) selective reporting risk. This analysis recorded pertinent data, including the first author, published year, study type, the sample size in CR and PS groups, outcome measurements, etc.

### Outcome measures

The outcome measurements included the kinematic gait parameters (flexion at heel strike, maximum flexion during loading response, flexion range during loading, minimal flexion at terminal stance, maximal flexion at the swing, and total flexion during the gait cycle), Knee Society Score, knee flexion, knee extension, and walking speed.

### Statistical analysis

All data analyses were performed with Stata 14.0 and Review Manager 5.4 statistical software provided by The Nordic Cochrane Centre. Continuous outcomes were expressed as the standard mean difference (SMD) or weighted mean difference (WMD), and relative risk (RR) or odds ratios (OR) were used for dichotomous outcomes and both with 95% confidence intervals (CIs). Heterogeneity was determined by estimating the proportion of between-study inconsistencies due to actual differences between studies, rather than differences due to random error or chance [15]. Both the chi-square test and *I*^2^ test were used to test heterogeneity. A fixed-effect model was adopted when there was no statistical evidence of heterogeneity (*P* > 0.10, *I*^2 ^< 50%) and a random effect model was chosen if significant heterogeneity was found (*P *≦ 0.10, *I*^2 ^≧ 50%) [16]. We checked the study population, methodology, treatments, and outcomes to determine the source when there was heterogeneity found. We used a qualitative evaluation if the heterogeneity could not be quantitatively synthesized or the event rate was too low to be measured. Besides, by excluding individual studies that caused heterogeneity for sensitivity analysis and making funnel plots to estimate the bias, which difference was statistically significant at *P* < 0.05.

## Results

### Search and selection

**Fig. 1:**
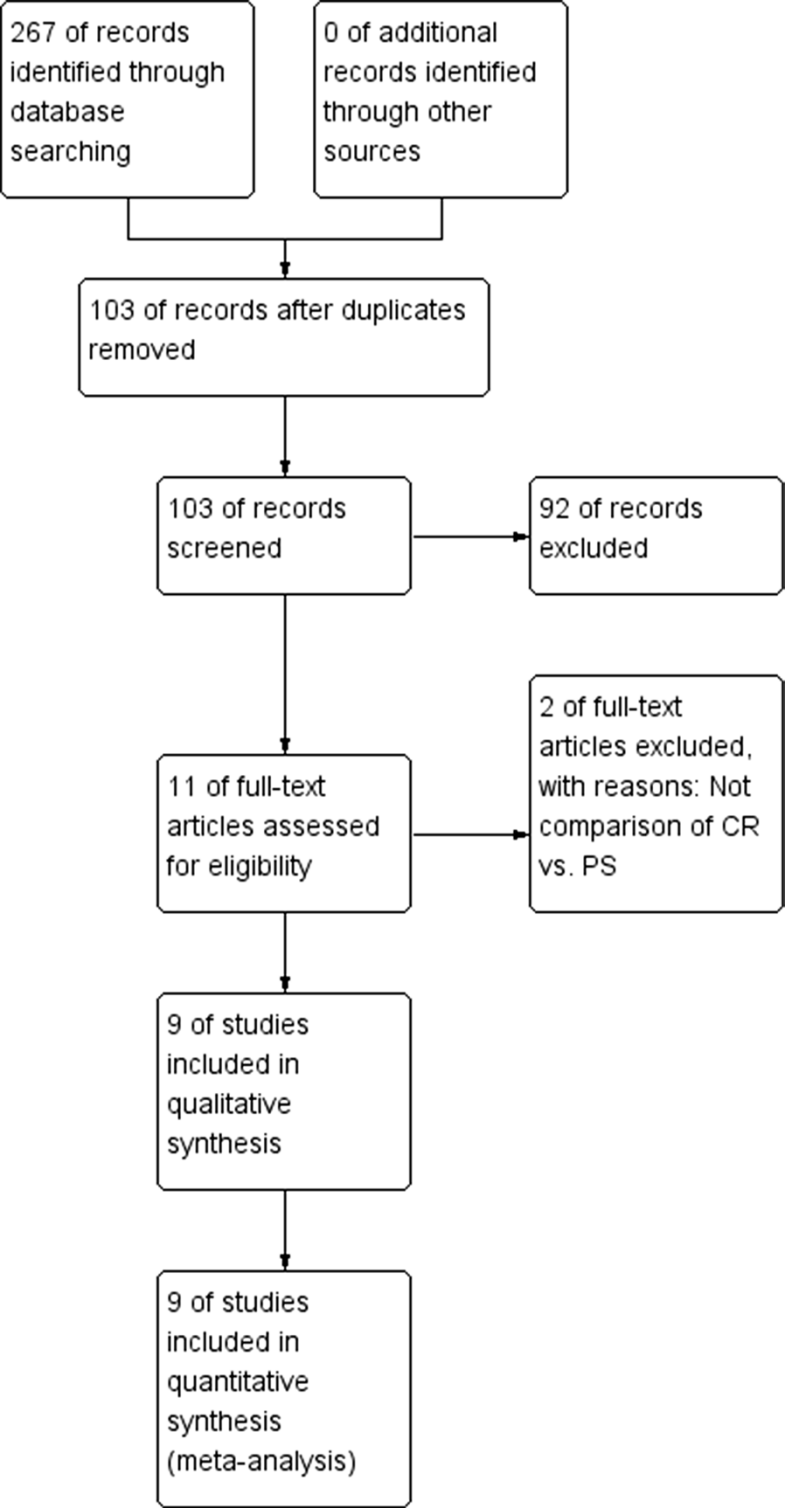
The flow diagram of searching studies

Via our online and manual searching, we initially identified 267 unique studies. After removing 164 duplicates, 103 studies remained. Of these, 92 were excluded after reading the titles and abstracts. The full texts were read, an additional two studies were excluded because they had unusable information or not appropriate comparison between CR and PS. Ultimately, 9 studies involving 351 knees that underwent gait analysis were included [13, 17–24]. The process and results of literature screening are shown in Fig. 1. One study compared retrospectively measured parameters, while the other eight studies compared parameters prospectively. Five studies compared groups according to knee flexion, and Knee Society Score; four compared knee extension, and maximum flexion during loading response; three compared walking speed, flexion range during loading, and maximal flexion at swing; and two compared flexion at heel strike, minimal flexion at terminal stance, and total flexion during the gait cycle. The basic characteristics and conditions of the nine studies included in this meta-analysis are presented in Table 1.

**Table 1:** Details of the included studies

First author	Year	Study type	Mean age (years)		Sample size (M/F)		Weight(kg)/height(m)		Follow-up (months)	Outcomes
			CR	PS	CR	PS	CR	PS		
Beach [21]	2019	PCS	67.6	69.1	26 (NA)	26 (NA)	85.3/1.696	89.8/1.711	At least 12	WS, F1
Bolanos [17]	1998	PCS	66	66	14 (1/13)	14 (1/13)	NA	NA	Mean 98	F2, F3, F5, F6, KSS
Gray [20]	2020	PCS	71.5	66.8	25 (16/9)	23 (14/9)	89.6/1.7	89.4/1.703	Mean 6	WS, F5, F6
Hajduk [13]	2016	PCS	68.13	65.68	23 (4/19)	19 (5/14)	84.43/1.63	87.42/1.65	At least 12	WS, F1, F2, F3, F4, F5, F6, F, KSS
Hamai [22]	2015	PCS	70	75	12 (1/11)	12 (0/12)	NA/1.51	NA/1.47	Mean 25	F, E, KSS
Ishii [18]	1998	PCS	67.6	68.5	11 (6/5)	9 (4/5)	NA	NA	At least 18	F2, F5
Maruyama [24]	2004	PCS	74.3	74.3	20 (8/12)	20 (8/12)	NA	NA	At least 24	F, E, KSS
Udomkiat [19]	2000	RCS	70.2	70.8	38 (16/22)	38 (16/22)	71.53/NA	71.67/NA	At least 24	F, E, KSS
van den Boom [23]	2014	PCS	72	75	9 (7/2)	12 (5/7)	NA	NA	At least 6	F1, F2, F3, F4, F, E, KSS

*RCS* retrospective comparative study, *PCS* prospective comparative study, *M* male, *F* female, *CR* posterior cruciate retention, *PS* posterior cruciate substitution, *NA* not available, *F1* flexion at heel strike (°), *F2* maximum flexion during loading response (°), *F3* flexion range during loading (°), *F4* minimal flexion at terminal stance (°), *F5* maximal flexion at swing (°), *F6* total flexion during the gait cycle (°), *F* knee flexion angle (°), *E* knee extension angle (°), *WS* walking speed (m/s), *KSS* Knee Society Score

**Fig. 2:**
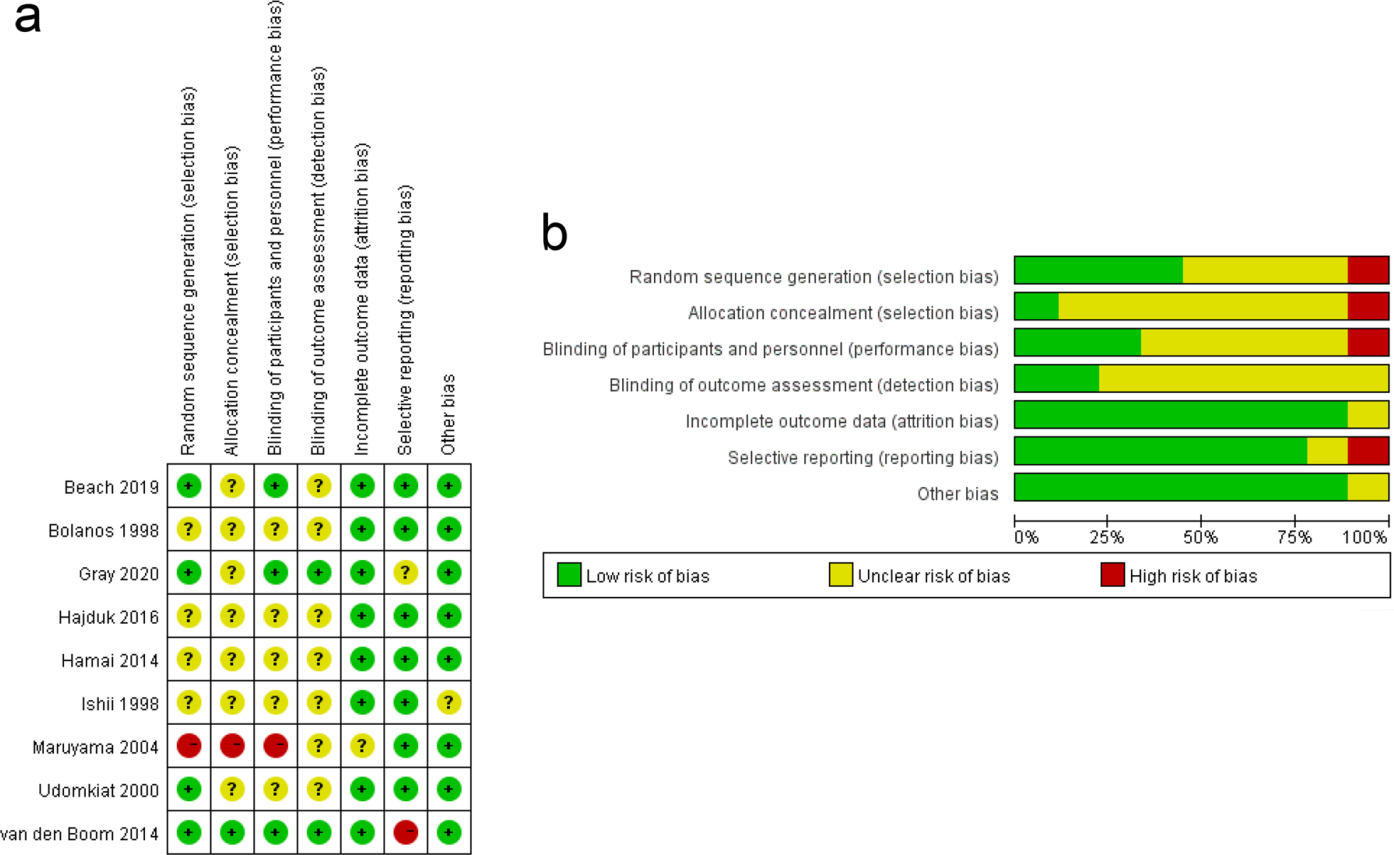
**a** Risk-of-bias graph of the included studies. The risk-of-bias tool includes the selection bias, performance bias, detection bias, attrition bias, reporting bias, and other bias. + , low risk; −, high risk; ?, unclear risk. **b** Risk-of-bias summary. In the included studies, each bias project is presented as a percentage, and the proportion level of each bias project is pointed out

Nine studies were included in the meta-analysis, and the heterogeneity among the outcomes of the included studies was estimated by the chi-square test and *I*^2^ test. The methodological quality of included studies was high, and the possibility of bias was low. The risk of bias summary and graph are respectively shown in Fig. 2.

### Kinematic gait parameters

**Fig. 3:**
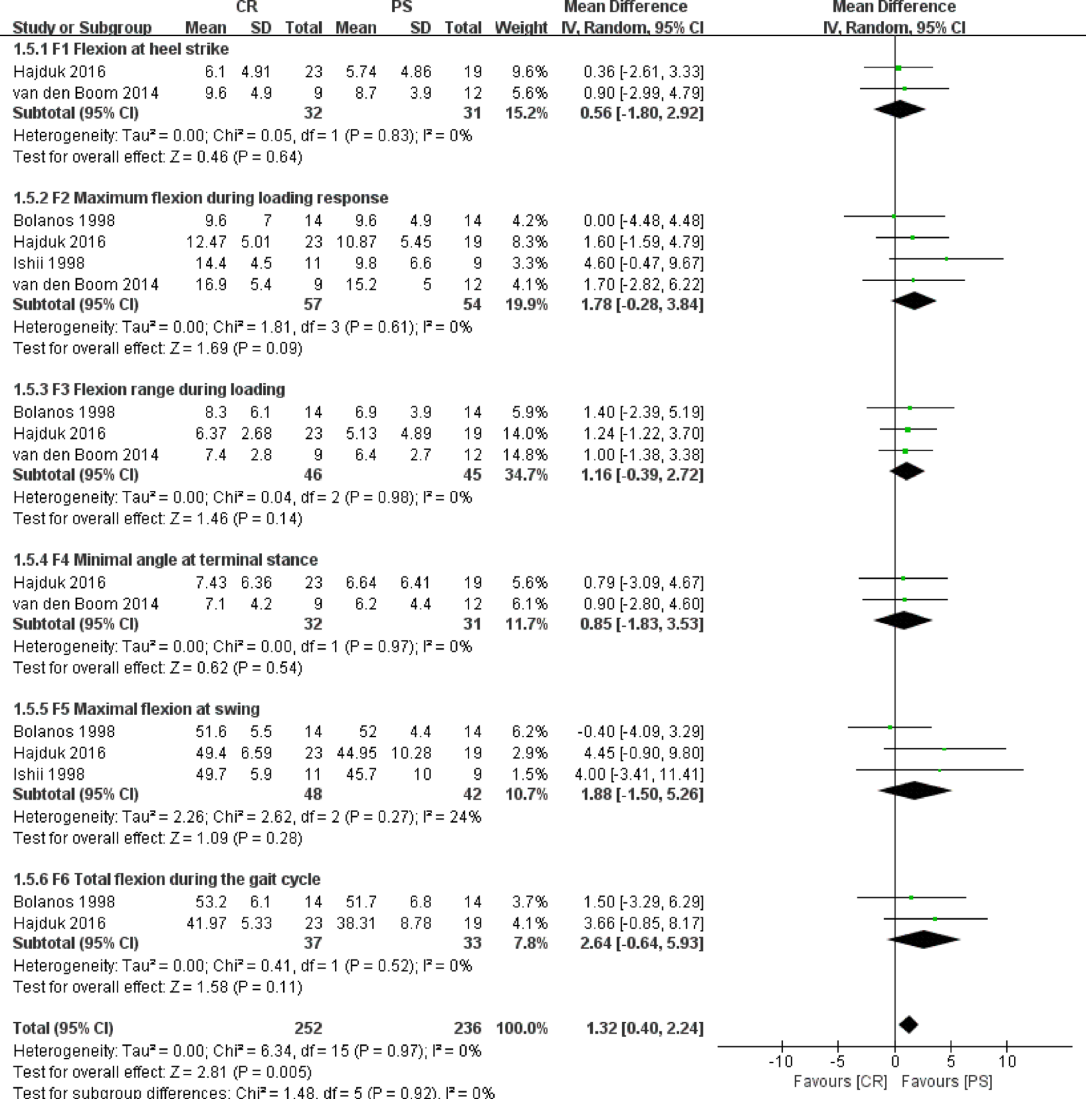
Results of aggregate analysis for comparison of kinematic gait parameters between CR and PS designs

Of the nine studies, the kinematic gait parameters including flexion at heel strike, maximum flexion during loading response, flexion range during loading, minimal flexion at terminal stance, maximal flexion at the swing, and total flexion during the gait cycle between CR and PS groups were compared in four studies involving 111 knees. Because all kinematic gait parameters above were continuous variables, the results were presented as mean and standard deviation. Flexion at heel strike between CR and PS groups was compared in two studies involving 63 knees. Fixed effect model was employed in this meta-analysis without heterogeneity (*P* = 0.83, *I*^2^ = 0%) between the two studies. Results showed that flexion at heel strike was greater in the CR group than that in the PS group, but the difference was not statistically significant (WMD = 0.56, 95% CI − 1.80 to 2.92, *P* = 0.64, Fig. 3).

Maximum flexion during loading response between CR and PS groups was compared in four studies involving 111 knees. Fixed effect model was employed in this meta-analysis without heterogeneity (*P* = 0.61, *I*^2^ = 0%) among the four studies. The results showed that maximum flexion during loading response was greater in the CR group than that in the PS group, but the difference was not statistically significant (WMD = 1.78, 95% CI − 0.28 to 3.84, *P* = 0.09, Fig. 3).

Flexion range during loading between CR and PS groups was compared in three studies involving 91 knees. Fixed effect model was employed in this meta-analysis without heterogeneity (*P* = 0.98, *I*^2^ = 0%) among the three studies. The results showed that flexion range during loading was greater in the CR group than that in the PS group, but the difference was not statistically significant (WMD = 1.16, 95% CI − 0.39 to 2.72, *P* = 0.14, Fig. 3).

Minimal flexion at terminal stance between CR and PS groups was compared in two studies involving 63 knees. Fixed effect model was employed in this meta-analysis without heterogeneity (*P* = 0.97, *I*^2^ = 0%) between the two studies. Results showed that minimal flexion at terminal stance was greater in the CR group than that in the PS group, but the difference was not statistically significant (WMD = 0.85, 95% CI − 1.83 to 3.53, *P* = 0.54, Fig. 3).

Maximal flexion at swing between CR and PS groups was compared in three studies involving 90 knees. Fixed effect model was employed in this meta-analysis without heterogeneity (*P* = 0.27, *I*^2^ = 24%) among the three studies. The results showed that maximal flexion at swing was greater in the CR group than that in the PS group, but the difference was not statistically significant (WMD = 1.57, 95% CI − 1.24 to 4.38, *P* = 0.27, Fig. 3).

Total flexion during the gait cycle between CR and PS groups was compared in two studies involving 70 knees. Fixed effect model was employed in this meta-analysis without heterogeneity (*P* = 0.52, *I*^2^ = 0%) between the two studies. Results showed that total flexion during the gait cycle was greater in the CR group than that in the PS group, but the difference was not statistically significant (WMD = 2.64, 95% CI − 0.64 to 5.93, *P* = 0.11, Fig. 3).

### Knee Society Score

**Fig. 4:**
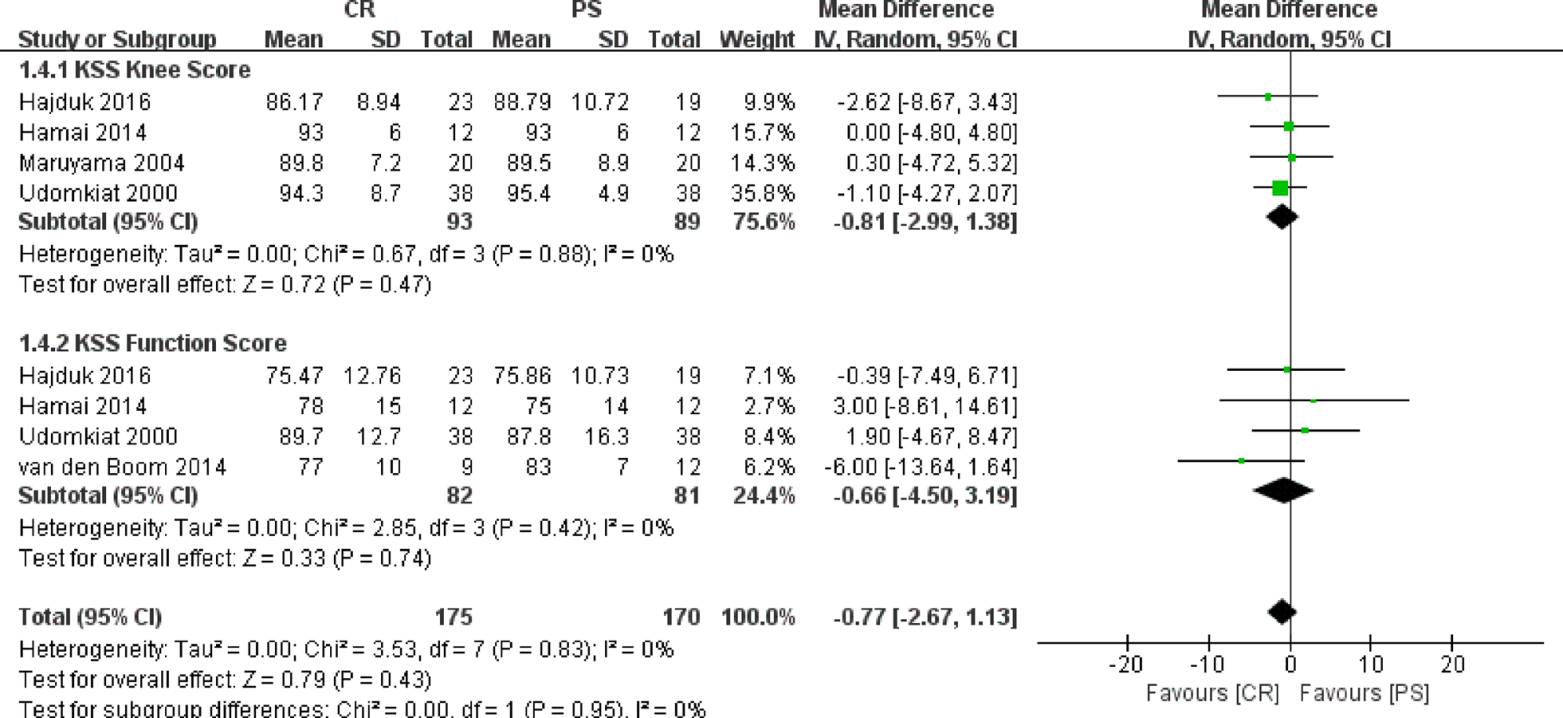
Results of aggregate analysis for comparison of KSS, including KSS Knee score and KSS Function score between CR and PS designs

Of the nine studies, Knee Society Score between CR and PS groups was compared in five studies involving 203 knees. Because Knee Society Score was a continuous variable, the results were presented as mean and standard deviation. The Knee Society Score was divided into two parts in terms of the KSS Knee score and KSS Function score. KSS Knee scores between CR and PS groups were compared in four studies involving 182 knees. Fixed effect model was employed in this meta-analysis without heterogeneity (*P* = 0.88, *I*^2^ = 0%) among the four studies. Results showed that the CR group had a lower KSS Knee score than that in the PS group, but the difference was not statistically significant (WMD = − 0.81, 95% CI − 2.99 to 1.38, *P* = 0.47, Fig. 4). Likewise, KSS Function scores between CR and PS groups were compared in four studies involving 163 knees. Fixed effect model was employed in this meta-analysis without heterogeneity (*P* = 0.42, *I*^2^ = 0%) among the four studies. The results showed that the CR group had a lower KSS Function score than that in the PS group, but the difference was also not statistically significant (WMD = − 0.66, 95% CI − 4.50 to 3.19, *P* = 0.74, Fig. 4).

### Knee flexion

**Fig. 5:**
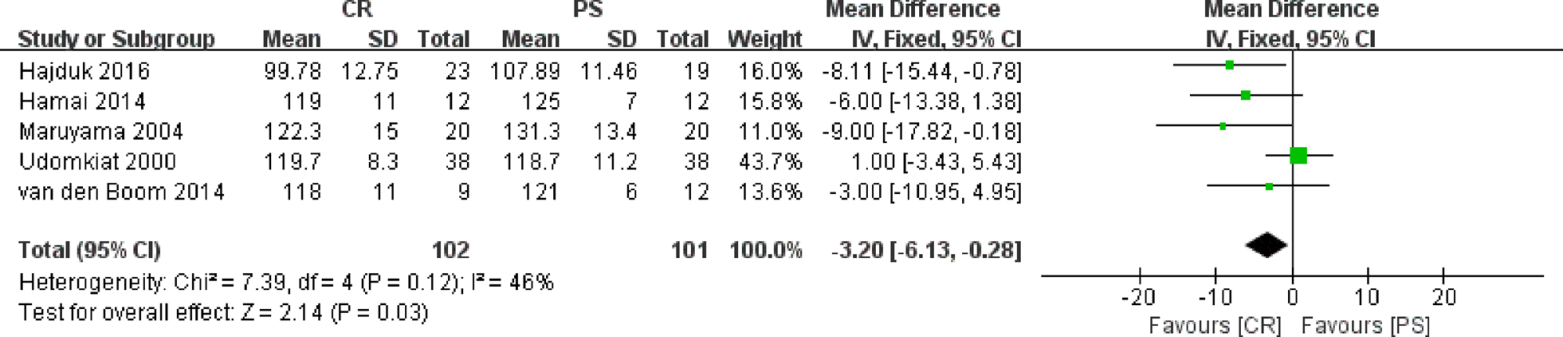
Results of aggregate analysis for comparison of knee flexion angle between CR and PS designs

Five studies involving 203 knees compared the knee flexion between posterior cruciate retention and substitution. Because knee flexion was a continuous variable, the results were presented as mean and standard deviation. Fixed effect model was employed in this meta-analysis without heterogeneity (*P* = 0.12, *I*^2^ = 46%) among the five studies. The results showed that the CR group had a significantly lower knee flexion angle than that in the PS group (WMD = − 3.20, 95% CI − 6.13 to − 0.28, *P* = 0.03, Fig. 5).

### Knee extension

**Fig. 6:**
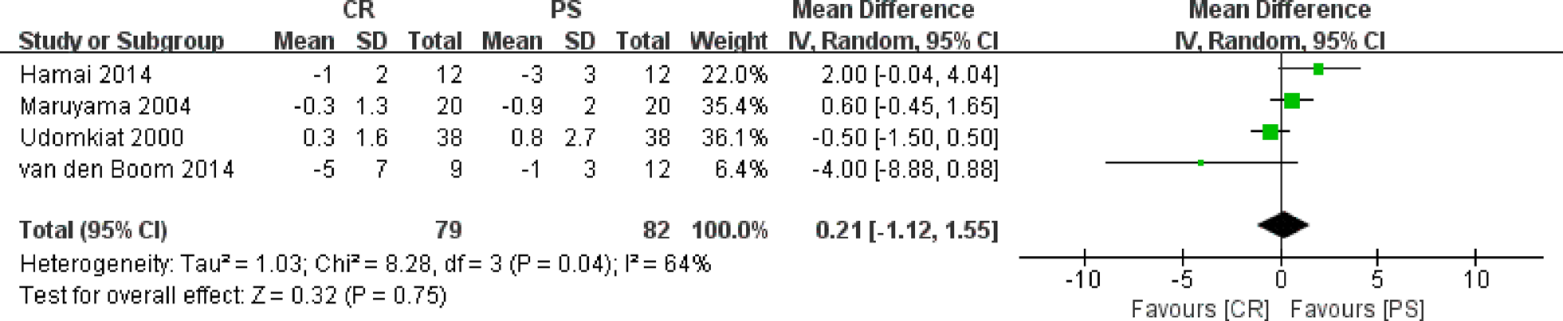
Results of aggregate analysis for comparison of knee extension angle between CR and PS designs

Four studies involving 161 knees compared the knee extension between posterior cruciate retention and substitution. Because knee extension was a continuous variable, the results were presented as mean and standard deviation. A random-effect model was employed in this meta-analysis because the heterogeneity among the studies was significant (*P* = 0.04, *I*^2^ = 64%). The meta-analysis showed that the knee extension angle was significantly smaller in the PS group than that in the CR group (WMD = 0.21, 95% CI − 1.12 to 1.55, *P* = 0.75, Fig. 6). There was no significant difference between the results of the sensitivity analysis and the initial analysis, which indicated that the findings strongly supported the decisions made in the process of obtaining them (Table 2).

**Table 2:** Sensitivity analysis

Study	Parameter	Before exclusion	After exclusion	Statistical significance
Hamai 2014	Knee extension angle	WMD = 0.21, 95% CI − 1.12 to 1.55, *Z* = 0.32, *P* = 0.75	WMD = − 0.23, 95% CI − 1.55 to 1.10, *Z* = 0.33, *P* = 0.74	No difference
Maruyama 2004			WMD = − 0.14, 95% CI − 2.53 to 2.25, *Z* = 0.12, *P* = 0.91	No difference
Udomkiat 2000			WMD = 0.50, 95% CI − 1.52 to 2.52, *Z* = 0.49, *P* = 0.63	No difference
van den Boom 2014			WMD = 0.46, 95% CI − 0.75 to 1.67, *Z* = 0.75, *P* = 0.45	No difference

*WMD* weighted mean difference, *CI* confidence interval

### Walking speed

**Fig. 7:**
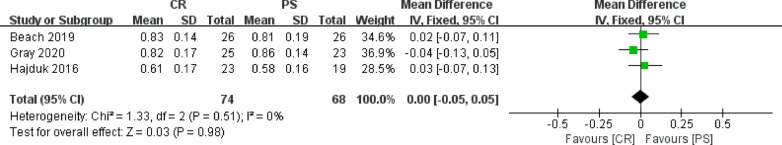
Results of aggregate analysis for comparison of walking speed between CR and PS designs

Three studies involving 142 knees compared the walking speed between posterior cruciate retention and substitution. Because walking speed was a continuous variable, the results were presented as mean and standard deviation. Fixed effect model was employed in this meta-analysis without heterogeneity (*P* = 0.51, *I*^2^ = 0%) among the three studies. The results showed that the walking speed in the CR group is the same as that in the PS group (WMD = 0.00, 95% CI − 0.05 to 0.05, *P* = 0.98, Fig. 7).

### Meta-regression analysis

As shown in Table 3, we summarized the results of the meta-regression analysis. Both gender and follow-up time was not significantly associated with the mean difference in knee extension angle, which indicated that both of them did not affect the mean difference in knee extension angle between CR and PS designs.

**Table 3:** Meta-regression analysis of follow-up time and gender in knee extension angle between CR and PS designs

Variable	Coefficient	Standard error	*P* value	95% confidence interval
Follow-up	0.177	0.152	0.453	− 1.760 to 2.114
Gender	− 5.095	3.509	0.384	− 49.676 to 39.486
Constant	− 2.131	4.264	0.705	− 56.305 to 52.043

## Discussion

Total knee arthroplasty is one of the most successful and effective operations for the treatment of end-stage knee osteoarthritis. Compared with the traditional TKA procedure, Aletto et al. [25] find that computer assisted TKA allows reproducible alignment and kinematics, reduces outliers, provides ligament balancing, and ensures good short-term postoperative functional outcomes. And Rossi et al. [26] also note the great clinical outcomes at mid-term follow up by the tensioner technique based on computer performing a ligament driven coronal alignment in TKA. At present, studies have shown that the knee prosthesis with medialized keel has good bone fixation and component alignment at a medium- to long-term follow-up [27], but the most widely used prostheses for TKA are still CR prosthesis and PS prosthesis in the unrestricted prosthesis. In the process of the development of knee prostheses, the advantages and disadvantages of two prostheses have been debated all the time. So the main purpose of this meta-analysis is to compare the gait of CR prosthesis and PS prosthesis in TKA, including the kinematic gait parameters, Knee Society Score, knee flexion, knee extension, and walking speed, to explore the effect of preservation of posterior cruciate ligament on gait after TKA, and to provide a clinical reference for the selection of prosthesis in TKA.

A good range of motion (ROM) of the joint is a necessary condition to ensure that people meet the movement of daily life. Install et al. [28] found that the average range of motion of the knee joint was 60° when walking on the flat ground, and at least 90° when walking up and down the stairs. In our study, the postoperative flexion angle of the knee joint was more than 90°, the postoperative pain was relieved and the quality of life was improved significantly. Besides, the range of motion of the knee joint after TKA is affected by various preoperative, intraoperative, and postoperative factors. Some studies have shown that the preoperative knee flexion is the main factor affecting the range of motion after TKA, whereas the patients with higher body mass index bear more weight and greater soft tissue resistance, which leads to the decrease of knee flexion after operation [29]. The posterior tibial osteotomy inclination angle also affects the range of motion of the knee joint after TKA. Related studies showed that when the angle of posterior tibial osteotomy inclination increased by 1°, the ROM of the knee joint increased by 1.7° [30]. Clinically, the posterior inclination of the tibia is usually 5°–7°, which is beneficial to increase the flexion of the knee joint after the operation. In addition, the degree of preoperative knee deformity, intraoperative soft tissue balance, postoperative pain, and functional exercise all affect the range of motion of the knee joint. Because of the paucity of other articles discussing similar information, none of these are included in statistical analysis.

The two kinds of prostheses complete the femoral roll movement through different mechanisms to achieve knee flexion. The CR prosthesis achieves normal femoral roll movement by preserving the posterior cruciate ligament so that the ROM of the knee joint is larger and the gait tends to be more normal after TKA. On the other hand, the PS prosthesis rolls back through the femoral cam device. When the knee flexion is about 70°, the femoral cam presses against the central column of the tibial prosthesis to move the contact point between the tibia and the femur backward, thus completing the femoral roll movement [31]. Several studies have shown that PS prosthesis is superior to CR prosthesis in knee flexion and ROM after TKA [11, 12]. Our study also showed that the mean difference of knee flexion angle was − 3.20° less in the CR group than PS group. And by studying the mechanism of knee flexion limitation after CR prosthesis TKA, Bellemans et al. [32] found that the main reason was the decrease of posterior condylar offset (PCO) after TKA and the occurrence of anterior rolling during knee flexion.

There are many kinds of methods to evaluate the curative effect after TKA, and we adopt the Knee Society Score in this study. The KSS, consisting of KSS Knee score and KSS Function score, is a comprehensive knee scoring standard put forward by the American knee society in 1989, comprehensively evaluating the overall function and morphology of the knee joint. And it can not only significantly detect the wear and tear of artificial joints with the increase of years, but also play a certain role in guiding patients' rehabilitation and functional exercise. Although many articles’ results show that the flexion of the knee joint after PS prosthesis is better than that of CR, there is no significant difference in KSS [1, 12, 33, 34], which is consistent with our study. It may be because the KSS is a comprehensive clinical score in which the ROM item has been covered.

Walking speed is one of the most significant parameters for evaluating functional outcomes after TKA and a small increase in walking speed may change the prognosis [35, 36]. Nevertheless, the walking speed of patients treated with TKA is still controversial. Theoretically, the CR group is greater in the working speed than the PS group, resulting from preserving posterior cruciate ligament playing an important role in proprioceptive joint control. Interestingly, our study did not support that one is over the other, because except for one parameter, including walking speed, there was no difference between the two groups. But the CR group retains the proprioceptive sensation and bone mass, which increases postoperative satisfaction of patients and provides an anatomical basis for future renovations [37, 38].

**Fig. 8:**
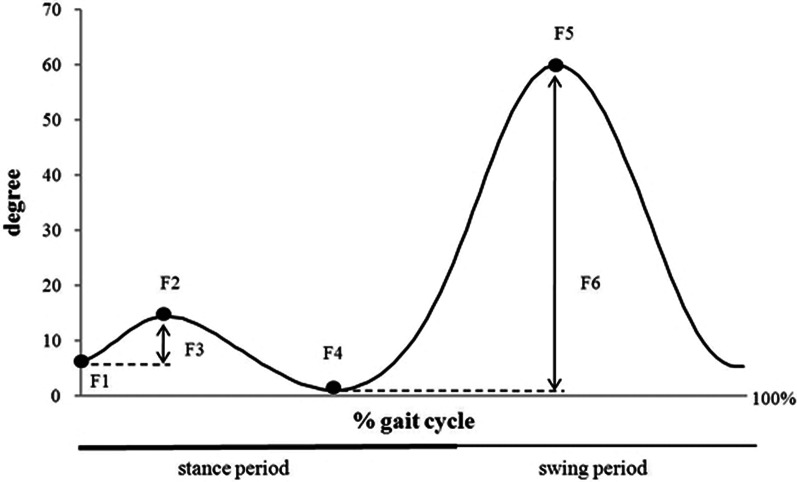
Peak values of knee flexion and extension at defined points of the gait cycle. F1—flexion at heel strike, F2—maximum flexion during loading response, F3—flexion range during loading, F4—minimal flexion at terminal stance, F5—maximal flexion at swing, F6—total flexion during the gait cycle

The peak values of knee flexion and extension at defined points of the gait cycle are shown in Fig. 8 [13]. Some studies found that knee flexion during the stance and swing phase was positively correlated with walking speed, which indicated that increased knee flexion angle at the stance phase for faster-walking speed may allow an even distribution of knee forces over a wider region of tibiofemoral cartilage [39]. However, our study showed that knee flexion angle in both groups did not show a significant difference during the two phases. There was a trend toward the CR group having a greater knee flexion angle than that in the PS group, whereas with the same walking speed. The discrepancies between the two groups may result from the sample size being not large enough.

Although there are some discoveries revealed by this study, there are also several limitations. First, this study did not compare preoperative gait parameters of patients, which could be associated with the postoperative gait difference between the two groups. Second, the use of various prosthetic materials and gait analysis systems were different among the included studies, which might bias the assessment of gait parameters. Third, we did not accurately assess the stair-climbing ability, stability, bone mass, the assessment of proprioception, and the effect of age and BMI on the outcome after TKA. Fourth, we conducted sensitivity analysis and meta-regression analysis of parameter knee extension angle, but we did not find the cause of the high heterogeneity. And the subgroup analysis was not conducted because the sample size was not large enough. Lastly, we included up to five studies and at least two studies for anyone outcome, which lead to the heterogeneity between groups increasing. Therefore, the above conclusions still need to be further verified, depending on whether there will be more randomized controlled trials with higher quality and larger sample sizes in the future.

## Conclusion

On the gait analysis, the CR design had a significantly lower knee flexion angle than that in PS design, but no significant difference was found in overall kinematic gait parameters, knee extension, walking speed, or Knee Society Score between CR and PS designs during level walking, which suggested that surgeons do not necessarily need a PS design to substitute the PCL during TKA. Additionally, more high-quality randomized controlled trials are needed to report comparable data on the clinical characteristics of total knee arthroplasty such as stability, pain relief, stair-climbing ability, bone mass, proprioception, etc.

## Data Availability

All data generated or analyzed during this study are included in this published article.
